# Supplementary material for: Dynamic predictive coding: A model of hierarchical sequence learning and prediction in the neocortex
Source: PLoS Comput Biol. 2024 Feb 8;20(2):e1011801. doi: 10.1371/journal.pcbi.1011801 (PMC10880975; doi:10.1371/journal.pcbi.1011801)
Supplement: S1 Text — Fig A. Improvement on test set loss saturates as the number of transition matrices increases. (a) Test set loss as training proceeded. Shaded area denotes ±1 standard deviation computed over eight runs with random initialization for each K. K = 1 shows the performance of the single-layer model. (b) Best test loss as K increases. Error bars denote ±1 standard deviation. Fig B. Cue-triggered recall is cue-specific. Four examples of cue-specific sequence recall by the associative memory model after training on different sequences, when given the first frame as the cue. In each quadrant: top: the original image sequence; bottom: cue-triggered recall of the stored sequence. Fig C. Prediction error threshold robustly finds changes of dynamics. (a) The distribution of first-level prediction errors in the two-level DPC model on the Moving MNIST training set. The red dashed line denotes the threshold ρ = 0.73, where the cumulative density reaches 0.75. (b) Examples of input sequences in the test set. The red arrows mark time steps when the first-level prediction errors exceeded ρ, corresponding to changes in input dynamics. Table A. DPC generative model parameters and values. Table B. Optimizers and learning rates used for inference and learning in the DPC experiments. Here Δ denotes the difference in rt or rh from before and after the current iteration of gradient descent. Table C. Memory model parameters and values. Table D. Optimizers and learning rates used for inference and learning in the memory model. Here Δ denotes the difference in m from before and after the current iteration of gradient descent. Table E. Additional parameters and values for the three-level DPC model. Table F. Additional optimizers and learning rates used for inference and learning in the three-level DPC experiments. Algorithm A. Inference & learning process. Algorithm B. Inference & learning process for the three-level DPC model. (PDF) [file pcbi.1011801.s001.pdf]

Supplementary Information for

Dynamic Predictive Coding: A Model of Hierarchical Sequence Learning and Prediction in the Neocortex

Linxing Preston Jiang[1,2,3], Rajesh P. N. Rao[1,2,3],

1 Paul G. Allen School of Computer Science & Engineering, University of Washington, Seattle, Washington, United States of America
2 Center for Neurotechnology, University of Washington, Seattle, Washington, United States of America
3 Computational Neuroscience Center, University of Washington, Seattle, Washington, United States of America

Hypernetworks and neural gain modulation

A hypernetwork $\mathcal{H}_\phi$ is a neural network parameterized by synaptic weights and bias parameters ϕ that generates the parameters (weights and bias) of another "primary" neural network $\mathbf{f}$ [1]:

$$\theta = \mathcal{H}_\phi(\mathbf{z}) \tag{41}$$
$$\mathbf{y} = \mathbf{f}_\theta(\mathbf{x}), \tag{42}$$

where $\mathbf{z}$ and $\mathbf{x}$ are the inputs to the hypernetwork and the primary network, respectively, and $\mathbf{y}$ is the output of the primary network. Therefore, the responses of the primary network to the same input $\mathbf{x}$ can be different, depending on the parameters θ generated by the hypernet. The input $\mathbf{z}$ to the hypernet can be a top-down input, a task input or context, an external input, or even past inputs $\mathbf{x}$. The hypernet model thus provides a general framework for one neural network (hypernet) modulating the function being computed by another neural network (primary net).

A special case of the hypernet model corresponds to neural gain modulation – a phenomenon where external or internal drives modulate the input-output (I/O) relationship of a neuron [2, 3]. Typically, gain modulation is implemented through multiplicative (altering the slope of the I/O relationship) or additive (shifting the I/O relationship) mechanisms on either the input or the response [2, 4, 5]. These multiplicative and additive components can be seen as the output of the hypernetwork fed to a primary network with fixed parameters θ. For example, the recurrent network model proposed by Stroud et al. (Eq 1 in [6]) can be written as (following our notation)

$$\tau \frac{\mathrm{d}\mathbf{r}_t}{\mathrm{d}t} = -\mathbf{r}_t + \mathbf{V}\mathbf{f}(\mathbf{r}_t; \mathbf{e}), \tag{43}$$

where τ is the time constant, $\mathbf{r}_t$ is the neural activity vector at time t, $\mathbf{V}$ is the recurrent weight matrix, and $\mathbf{e}$ is the gain modulation that works as a pointwise multiplicative factor on the input ($f_i(r_i; e_i) \approx \tanh(e_i r_i)$, see Equation 2 in Ref. [6]). Here, $\mathbf{e}$ can be seen as the output of a hypernetwork $\mathcal{H}_\phi$ that modulates the recurrent network with weights $\theta = \mathbf{V}$.

Similarly, the DPC generative model for hierarchical temporal prediction through top-down modulation of recurrent connections (Equation 7-9) can be implemented using

Parameter	Description	Value (Natural/MNIST)
$\mathbf{r}_t \in \mathbb{R}^N \quad \forall t = 0 \dots T-1$	Lower-level latent variable from time 0 to $T-1$	$N = 512/648$
$\mathbf{r}^h \in \mathbb{R}^{N_h}$	Higher-level latent variable	$N_h = 20/20$
$\mathbf{I}_t \in \mathbb{R}^M \quad \forall t = 0 \dots T-1$	Input observations from time 0 to $T-1$	$M = 256/324$
$\mathbf{U} \in \mathbb{R}^{M \times N}$	Learnable spatial filters	Learned
$\sigma^2 \in \mathbb{R}$	Spatial variance	1 / 1
$\sigma_r^2 \in \mathbb{R}$	Temporal variance	0.4 / 0.4
K	Number of transition matrices	$K = 5/5$
$\mathbf{V}_k \in \mathbb{R}^{N \times N} \quad \forall k = 1 \dots K$	Transition matrices	Learned
$\mathcal{H}_\theta$	Top-down dynamics network	Learned
λ	Sparsity penalty for $\mathbf{r}_t$	$5 \times 10^{-4}/1 \times 10^{-3}$
λ_h	Prior penalty for $\mathbf{r}^h$	$5 \times 10^{-6}/5 \times 10^{-6}$

Table A. DPC generative model parameters and values.

the recurrent network model:

$$\mathbf{w} = \mathcal{H}_\phi(\mathbf{r}^h) \tag{44}$$

$$\tau \frac{\mathrm{d}\mathbf{r}_t}{\mathrm{d}t} = -\mathbf{r}_t + \sum_k w_k \mathbf{V}_k \,\mathrm{ReLU}(\mathbf{r}_t) \tag{45}$$

$$= -\mathbf{r}_t + \sum_k \mathbf{V}_k \mathbf{f}_k(\mathbf{r}_t; w_k). \tag{46}$$

where $\mathbf{f}_k(\mathbf{r}_t; w_k) = w_k \,\mathrm{ReLU}(\mathbf{r}_t)$ is the input modulation function which modulates the inputs $\mathrm{ReLU}(\mathbf{r}_t)$ to group k of neurons multiplicatively with the weight w_k. Therefore, comparing Eq 43 with Eq 46, the gain modulation model of Stroud et al. [6] uses *per-neuron* multiplicative gain modulation while DPC generates a *more diffuse* top-down multiplicative gain modulation targeting *groups of lower-level neurons*, with the same top-down modulation strength w_k for group k. Such a model for top-down modulation is consistent with previous work suggesting that cortical feedback connections are more diffuse than feedforward connections [7–9] (but see also [10]).

Summary of the DPC generative model

Table A summarizes the parameters and values used in the DPC generative model. The top-down dynamics prediction network $\mathcal{H}$ (a hypernetwork) is a multi-layer perceptron (MLP) as follows. The first layer of the MLP contains 10 hidden neurons, followed by a LayerNorm layer [11] and an ELU activation function [12]. The last two layers of the MLP are comprised of (1) a linear layer with 10 hidden neurons, and (2) an output layer that maps this hidden activity to $\mathbf{w} \in \mathbb{R}^K$.

Inference and learning

Table B summarizes the optimizers and learning rates used for inference and learning in DPC. Algorithm A describes the inference (finding the MAP estimates of the latent variables) and learning (parameter estimation) process.

Training results

Fig Aa visualizes the evolution of test set loss as a function of training epochs. Besides results using five transition matrices ($K = 5$), we additionally trained models with

Optimizee / Other Hyperparameters	Optimizer (learning rate: Natural/MNIST) / Value
Inference	
$\mathbf{r}_t \quad \forall t = 0 \ldots T-1$	SGD (3/2.5)
$\mathbf{r}^h$	ADAM ($1 \times 10^{-3}/1 \times 10^{-3}$)
Convergence criteria	$\|\Delta\|_2 \leq 0.01$
Learning	
$\mathbf{U}$	SGD (2.5/0.5)
$\mathbf{V}_k \in \mathbb{R}^{N \times N} \quad \forall k = 1 \ldots K$	ADAM ($5 \times 10^{-4}/1 \times 10^{-3}$)
$\mathcal{H}_\theta$	ADAM ($5 \times 10^{-4}/1 \times 10^{-3}$)
Batch size	512/512
Epochs	100/120
Learning rate exponential decay	0.98 / 0.985

Table B. Optimizers and learning rates used for inference and learning in the DPC experiments. Here Δ denotes the difference in $\mathbf{r}_t$ or $\mathbf{r}^h$ from before and after the current iteration of gradient descent.

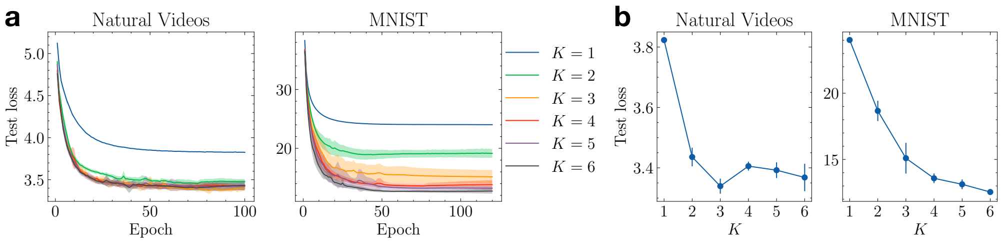

Fig A. Improvement on test set loss saturates as the number of transition matrices increases. (a) Test set loss as training proceeded. Shaded area denotes ± 1 standard deviation computed over eight runs with random initialization for each K. $K = 1$ shows the performance of the single-layer model. **(b)** Best test loss as K increases. Error bars denote ± 1 standard deviation.

$K = 1, \ldots, 6$, each with eight random initialization of weights. DPC with $K = 1$ is equivalent to a one-level Kalman filter model by [13] with an additional sparseness constraint on neural activities [14]. As shown in Fig A, all trained models converged after around 50 training epochs. DPC models with two-level architectures ($K > 1$) performed significantly better on the test sets. Such improvement in test set performance saturated as K increased. Fig Ab shows test set loss as a function of K for natural videos and the moving MNIST dataset.

Summary of the memory model

Table C summarizes the parameters and values used in the associative memory model. Table D summarizes the optimizers and learning rates used for inference and learning in the memory layer.

Fig B shows examples of cue-specific sequence recall by the associative memory model after training on different sequences. The upper left quadrant shows the image sequence used in our experiment in Fig 5, and the image sequence recalled by the trained memory-augmented DPC network. To test that the recall is cue-specific, we additionally conditioned the network with three sequences with different digits and dynamics. As the rest of Fig B shows, the memory-augmented DPC network successfully recalled the correct dynamics associated with the different digit cues,

Algorithm A Inference & learning process

Input: Image sequences dataset $\mathcal{D}$

Parameters: $\mathbf{U}, \{\mathbf{V}_k\}_{k=1}^{K}, \theta$

while the parameters have not converged **do**
    Sample minibatch $\{\mathbf{I}_{0:T-1}^{(m)}\}_{m=1}^{M}$ from dataset $\mathcal{D}$
    Initialize total loss $\mathcal{L} = 0$
    `// Inference`
    for each sequence $\mathbf{I}_{0:T-1}$ in the minibatch **do**
        Initialize $\mathbf{r}_0, \mathbf{r}^h$ as zero vectors
        $\hat{\mathbf{r}}_0 = \arg\min_{\mathbf{r}_0} \mathcal{L}_0(\mathbf{r}_0, \mathbf{U}, \mathbf{I}_0)$
        Update total loss $\mathcal{L} = \mathcal{L} + \mathcal{L}_0(\hat{\mathbf{r}}_0, \mathbf{U}, \mathbf{I}_0)$
        for $t = 1 \ldots T-1$ **do**
            Initialize $\mathbf{r}_t$ as a zero vector
            $\hat{\mathbf{r}}_t = \arg\min_{\mathbf{r}_t} \mathcal{L}_t(\mathbf{r}_t, \hat{\mathbf{r}}_{t-1}, \hat{\mathbf{r}}_h, \mathbf{U}, \{\mathbf{V}_k\}_{k=1}^{K}, \theta, \mathbf{I}_t)$
            $\hat{\mathbf{r}}^h = \arg\min_{\mathbf{r}^h} \mathcal{L}_t(\hat{\mathbf{r}}_t, \hat{\mathbf{r}}_{t-1}, \mathbf{r}_h, \mathbf{U}, \{\mathbf{V}_k\}_{k=1}^{K}, \theta, \mathbf{I}_t)$
            Update total loss $\mathcal{L} = \mathcal{L} + \mathcal{L}_t(\hat{\mathbf{r}}_t, \hat{\mathbf{r}}_{t-1}, \hat{\mathbf{r}}_h, \mathbf{U}, \{\mathbf{V}_k\}_{k=1}^{K}, \theta, \mathbf{I}_t)$
        end for
    end for
    `// Learning`
    Update parameters using gradients calculated from $\frac{1}{M}\mathcal{L}$ and their corresponding optimizers from Table B
end while

Return $\mathbf{U}, \{\mathbf{V}^{(k)}\}_{k=1}^{K}, \theta$

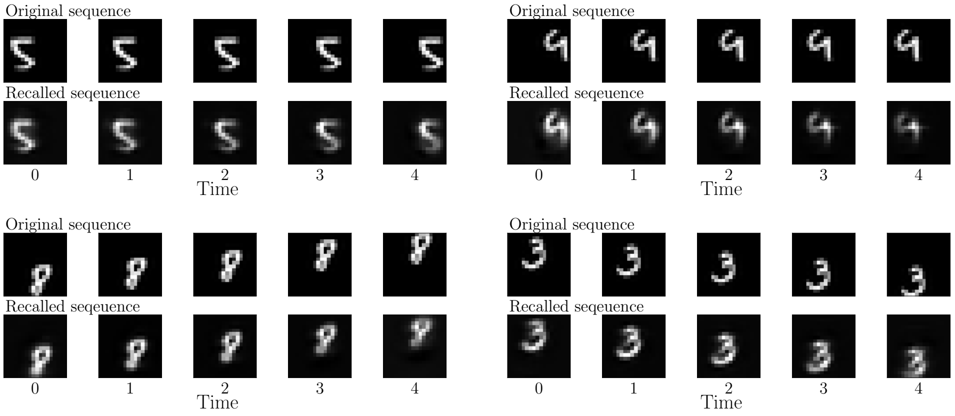

Fig B. Cue-triggered recall is cue-specific. Four examples of cue-specific sequence recall by the associative memory model after training on different sequences, when given the first frame as the cue. In each quadrant: top: the original image sequence; bottom: cue-triggered recall of the stored sequence.

demonstrating the cue-specificity of the network.

Parameter	Description	Value
$\mathbf{m} \in \mathbb{R}^{N_m}$	memory latent variable from time 1 to T	$N_m = 256$
$\mathbf{G} \in \mathbb{R}^{(N+N_h)\times N_m}$	Memory weights	Learned
λ_m	Prior penalty for $\mathbf{m}$	0

Table C. Memory model parameters and values.

Optimizee / Other Hyperparameters	Optimizer (learning rate) / Value
<u>Inference</u>	
$\mathbf{m}$	ADAM (0.005)
Convergence criteria	$\|\Delta\|_2 \leq 0.01$
<u>Learning</u>	
$\mathbf{G}$	ADAM (0.001)
Epochs	5
Learning rate exponential decay	0.99

Table D. Optimizers and learning rates used for inference and learning in the memory model. Here Δ denotes the difference in $\mathbf{m}$ from before and after the current iteration of gradient descent.

Three-level DPC model

We first describe the procedure for computing the threshold for detecting large first-level prediction errors. Using the pretrained two-level DPC model, we collect the distribution of all first-level prediction errors $\|\hat{\mathbf{r}}_t^{(1)} - \bar{\mathbf{r}}_t^{(1)}\|_2^2$ as defined in Equation 17. Fig Ca shows the distribution of prediction errors on the training set. We set the threshold $\rho = 0.73$ where the cumulative density of the error distribution (orange curve) reaches 0.75. To verify the efficacy of this threshold, in Fig Cb we show example input sequences from the test set, where the red arrows mark the time steps when the first-level prediction errors exceeded the threshold ρ. As shown, using this threshold robustly finds the moments when the input dynamics changed, in both straight bouncing sequences and clockwise bouncing sequences.

Table E describes the additional parameters and values in the three-level DPC model, in addition to those shown in Table A for the two-level model. The third-level top-down dynamics prediction network $\mathcal{H}_\theta^{(2)}$ is a multi-layer perceptron that is identical to the previously described second-level network $\mathcal{H}_\theta^{(1)}$, except the last output layer that has output dimension $K^{(2)} = 2$. Table F summarizes the optimizers and learning rates used for inference and learning in the three-level DPC experiments. Lastly, we describe the inference and learning process of the three-level model in Algorithm B.

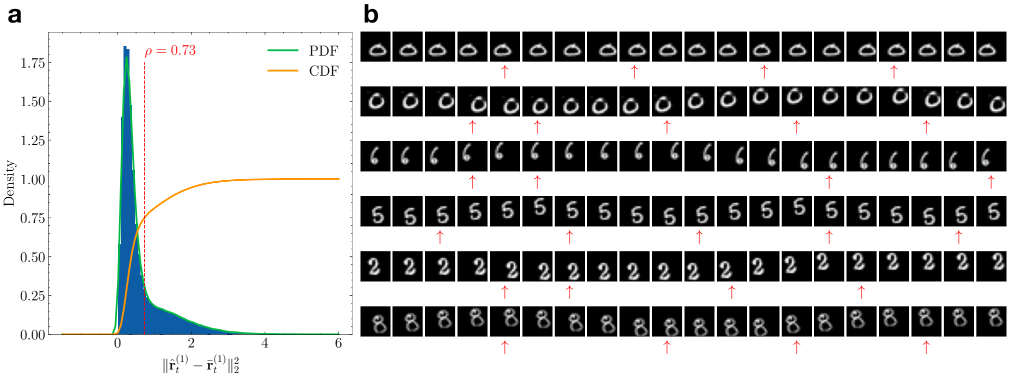

Fig C. Prediction error threshold robustly finds changes of dynamics.(a) The distribution of first-level prediction errors in the two-level DPC model on the Moving MNIST training set. The red dashed line denotes the threshold $\rho = 0.73$, where the cumulative density reaches 0.75. **(b)** Examples of input sequences in the test set. The red arrows mark time steps when the first-level prediction errors exceeded ρ, corresponding to changes in input dynamics.

Parameter	Description	Value
$\mathbf{r}^{(3)} \in \mathbb{R}^{N^{(3)}}$	Third-level latent variable	$N^{(3)} = 10$
$\sigma^2_{r^{(2)}} \in \mathbb{R}$	Variance of second-level prediction error	1
$K^{(2)}$	Number of second-level transition matrices	2
$\mathbf{V}_k^{(2)} \in \mathbb{R}^{N^{(2)} \times N^{(2)}} \quad \forall k = 1 \dots K^{(2)}$	Learnable second-level transition matrices	Learned
$\mathcal{H}_\theta^{(2)}$	Third-level top-down dynamics network	Learned

Table E. Additional parameters and values for the three-level DPC model.

Optimizee / Criteria	Optimizer / Value
Inference	
$\mathbf{r}_t^{(2)} \quad \forall t = 1 \dots T-1$	ADAM (1×10^{-3})
$\mathbf{r}^{(3)}$	ADAM (1×10^{-3})
Convergence criterion	$\|\Delta\|_2 \leq 0.01$
Learning	
$\mathbf{V}_k \in \mathbb{R}^{N^{(2)} \times N^{(2)}} \quad \forall k = 1 \dots K^{(2)}$	ADAM (1×10^{-3})
$\mathcal{H}_\theta^{(2)}$	ADAM (1×10^{-3})

Table F. Additional optimizers and learning rates used for inference and learning in the three-level DPC experiments.

Algorithm B Inference & learning process for the three-level DPC model

Input: Image sequences dataset $\mathcal{D}$
Parameters: $\mathbf{U}, \{\mathbf{V}_k^{(1)}\}_{k=1}^{K^{(1)}}, \{\mathbf{V}_k^{(2)}\}_{k=1}^{K^{(2)}}, \theta$, ρ (the first-level error threshold)

while the parameters have not converged **do**
    Sample minibatch $\{\mathbf{I}_{0:T-1}^{(m)}\}_{m=1}^{M}$ from dataset $\mathcal{D}$
    Initialize total loss $\mathcal{L} = 0$
    `// Inference`
    for each sequence $\mathbf{I}_{0:T-1}$ in the minibatch **do**
        Initialize $\mathbf{r}_0^{(1)}, \mathbf{r}_0^{(2)}, \mathbf{r}^{(3)}$ as zero vectors
        $\hat{\mathbf{r}}_0^{(1)} = \arg\min_{\mathbf{r}_0^{(1)}} \mathcal{L}_0(\mathbf{r}_0, \mathbf{U}, \mathbf{I}_0)$ ▷ Eq 20
        Update total loss $\mathcal{L} = \mathcal{L} + \mathcal{L}_0(\hat{\mathbf{r}}_0^{(1)}, \mathbf{U}, \mathbf{I}_0)$
        $\hat{\mathbf{r}}_1^{(1)}, \hat{\mathbf{r}}_1^{(2)} = \arg\min_{\mathbf{r}_1^{(1)}, \mathbf{r}_1^{(2)}} \mathcal{L}_1(\mathbf{r}_1^{(1)}, \mathbf{r}_1^{(2)}, \hat{\mathbf{r}}_0^{(1)}, \mathbf{U}, \{\mathbf{V}_k^{(1)}\}_{k=1}^{K^{(1)}}, \theta, \mathbf{I}_1)$ ▷ Eq 17
        Update total loss $\mathcal{L} = \mathcal{L} + \mathcal{L}_1(\hat{\mathbf{r}}_1^{(1)}, \hat{\mathbf{r}}_1^{(2)}, \hat{\mathbf{r}}_0^{(1)}, \mathbf{U}, \{\mathbf{V}_k^{(1)}\}_{k=1}^{K^{(1)}}, \theta, \mathbf{I}_1)$
        for $t = 2 \ldots T-1$ **do**
            `// Same two-level inference as before`
            $\hat{\mathbf{r}}_t^{(1)}, \hat{\mathbf{r}}_t^{(2)} = \arg\min_{\mathbf{r}_t^{(1)}, \mathbf{r}_t^{(2)}} \mathcal{L}_t(\mathbf{r}_t^{(1)}, \mathbf{r}_t^{(2)}, \hat{\mathbf{r}}_{t-1}^{(1)}, \mathbf{U}, \{\mathbf{V}_k^{(1)}\}_{k=1}^{K^{(1)}}, \theta, \mathbf{I}_t)$ ▷ Eq 17
            `// Compute binary mask`
            $b_t = \left(\|\hat{\mathbf{r}}_t^{(1)} - \bar{\mathbf{r}}_t^{(1)}\|_2^2 > \rho\right)$
            `// Eq 37`
            $\hat{\mathbf{r}}_t^{(2)}, \hat{\mathbf{r}}_t^{(3)} = \arg\min_{\mathbf{r}_t^{(2)}, \mathbf{r}^{(3)}} \mathcal{L}_t(\mathbf{r}_t^{(2)}, \mathbf{r}^{(3)}, \hat{\mathbf{r}}_{t-1}^{(1)}, \hat{\mathbf{r}}_{t-1}^{(2)}, b_t, \mathbf{U}, \{\mathbf{V}_k^{(1)}\}_{k=1}^{K^{(1)}}, \{\mathbf{V}_k^{(2)}\}_{k=1}^{K^{(2)}}, \theta, \mathbf{I}_t)$
            Update total loss $\mathcal{L} = \mathcal{L} + \mathcal{L}_t(\hat{\mathbf{r}}_t^{(2)}, \hat{\mathbf{r}}^{(3)}, \hat{\mathbf{r}}_{t-1}^{(1)}, \hat{\mathbf{r}}_{t-1}^{(2)}, b_t, \mathbf{U}, \{\mathbf{V}_k^{(1)}\}_{k=1}^{K^{(1)}}, \{\mathbf{V}_k^{(2)}\}_{k=1}^{K^{(2)}}, \theta, \mathbf{I}_t)$
        end for
    end for
    `// Learning`
    Update parameters using gradients calculated from $\frac{1}{M}\mathcal{L}$ and their corresponding optimizers from Table F
end while
Return $\{\mathbf{V}_k^{(2)}\}_{k=1}^{K^{(2)}}, \theta$

References

1. Ha D, Dai AM, Le QV. HyperNetworks. In: 5th International Conference on Learning Representations (ICLR 2017); 2017.

2. Ferguson KA, Cardin JA. Mechanisms underlying gain modulation in the cortex. Nature Reviews Neuroscience. 2020;21(2):80–92. doi:10.1038/s41583-019-0253-y.

3. Shine JM, Müller EJ, Munn B, Cabral J, Moran RJ, Breakspear M. Computational models link cellular mechanisms of neuromodulation to large-scale neural dynamics. Nature Neuroscience. 2021;24(6):765–776. doi:10.1038/s41593-021-00824-6.

4. Larkum ME, Senn W, Lüscher HR. Top-down Dendritic Input Increases the Gain of Layer 5 Pyramidal Neurons. Cerebral Cortex. 2004;14(10):1059–1070. doi:10.1093/cercor/bhh065.

5. Silver RA. Neuronal arithmetic. Nature Reviews Neuroscience. 2010;11(7):474–489. doi:10.1038/nrn2864.

6. Stroud JP, Porter MA, Hennequin G, Vogels TP. Motor primitives in space and time via targeted gain modulation in cortical networks. Nature Neuroscience. 2018;21(12):1774–1783. doi:10.1038/s41593-018-0276-0.
7. Perkel DJ, Bullier J, Kennedy H. Topography of the afferent connectivity of area 17 in the macaque monkey: A double-labelling study. Journal of Comparative Neurology. 1986;253(3):374–402. doi:10.1002/cne.902530307.
8. Salin PA, Bullier J. Corticocortical connections in the visual system: structure and function. Physiological Reviews. 1995;75(1):107–154. doi:10.1152/physrev.1995.75.1.107.
9. Rockland KS, Ojima H. Multisensory convergence in calcarine visual areas in macaque monkey. International Journal of Psychophysiology. 2003;50(1):19–26. doi:10.1016/S0167-8760(03)00121-1.
10. Markov NT, Vezoli J, Chameau P, Falchier A, Quilodran R, Huissoud C, et al. Anatomy of hierarchy: Feedforward and feedback pathways in macaque visual cortex. Journal of Comparative Neurology. 2014;522(1):225–259. doi:10.1002/cne.23458.
11. Ba JL, Kiros JR, Hinton GE. Layer Normalization; 2016. Available from: `http://arxiv.org/abs/1607.06450`.
12. Clevert DA, Unterthiner T, Hochreiter S. Fast and accurate deep network learning by exponential linear units (ELUs). In: International Conference on Learning Representations; 2016.Available from: `http://arxiv.org/abs/1511.07289`.
13. Rao RPN. An optimal estimation approach to visual perception and learning. Vision Research. 1999;39(11):1963–1989. doi:10.1016/S0042-6989(98)00279-X.
14. Olshausen BA, Field DJ. Emergence of simple-cell receptive field properties by learning a sparse code for natural images. Nature. 1996;381(6583):607–609. doi:10.1038/381607a0.
